# Supplementary material for: Molecular Insight into Iron Homeostasis of Acute Myeloid Leukemia Blasts
Source: Int J Mol Sci. 2023 Sep 19;24(18):14307. doi: 10.3390/ijms241814307 (PMC10531764; doi:10.3390/ijms241814307)
Supplement: Supplementary file 1 [file ijms-24-14307-s001.zip › ijms-2594398-supplementary.pdf]

Table S1. Variability of the expression of some genes of interest among CD34+ cells of healthy donors.

Gene (symbol)	Mean Relative Quantity to normalizers	CV%
Transferrin receptor (TFRC1)	0.31	52
Ferritin H (FTH1)	5.9	52
Lactate dehydrogenase (LDHA)	0.32	33
Hypoxia Inducible Factor 1α (HIF1a)	0.03	62

The coefficient of variation measured by qPCR for the listed genes is given for a set of $CD34^+$ cells from 4 cord blood samples, 3 bone marrow aspirates of adults undergoing cardiac surgery, and 2 samples of mobilized cells from healthy young adults. All expression levels were normalized to the three reference genes RPLP0, HPRT, and ACTB.

Table S2. Main characteristics of the newly diagnosed AML samples used in this study

**Gender**	**male (%)**	**female (%)**		
	19 (63.3)	11 (36.7)		
**Age (yr)**	**average**	**Range**		
	61.5	7-87		
**FAB classification**	**M0/M1/M2**	**M4/M5**		
number	20	10		
including < 20% $CD34^+$	9	6		
**Main genetic abnormalities**	**FLT3**	**WT1**	**NPM1**	**DNMT3A**
number	12	18	8	6
**Prognosis**	**Favorable**	**Intermediate**	**Adverse**	
number	4	18	8	

Table S3. Antibodies used in the present work.

Antigen	Provider	Cat. No
pan-Actin	Cell Signaling Technologies	D18C11 #8456
β-tubulin		9F3 #2128
HIF1α		D1S7W #36169
PDK1 (as PDHK1 at CST)		C47H1 #3820
CD71 (Transferrin Receptor 1)		D7G9X #13113
FTH1		D1D4 #4393
IRP1	Home made	[52]
IRP2	Home made	[26]
HIF2α (EPAS1)	Novus Biologicals<br>Bethyl Laboratories	NB100-122<br>A700-002-Tor A700-003

Table S4. Oligonucleotides (5’ -> 3’) used in qPCR experiments

Gene (encoded protein)	Forward and reverse primers
RPLP0 (ribosomal protein lateral stalk subunit P0)	GAAATCCTGGGTGTCCGCAATGTT<br>AGACAAGGCCAGGACTCGTTTGTA
HPRT1 (HPRT, hypoxanthine phosphoribosyltransferase 1)	ATGGACAGGACTGAACGTCTTGCT<br>TTGAGCACACAGAGGGCTACAATG
ACTB (β-actin)	GGATCAGCAAGCAGGAGTATG<br>AGAAAGGGTGTAACGCAACTAA
RRM2 (ribonucleotide reductase regulatory subunit M2)	CACGGAGCCGAAAACTAAAGC<br>TCTGCCTTCTTATACATCTGCCA
HIF1A (hypoxia inducible factor 1 subunit α)	ACGTGTTATCTGTCGCTTTGAGT<br>TCGTCTGGCTGCTGTAATAATGT
HIF2A (EPAS1 – endothelial PAS domain protein 1)	CGTCCTGAGTGAGATTGAGAAG<br>TCCTCCTTTAGCTTGGTGAATAG
HMOX1 (heme oxygenase 1)	CAGTGCCACCAAGTTCAAGC<br>GTTGAGCAGGAACGCAGTCTT
HMOX2 (heme oxygenase 2)	GGAGCGCAACAAGGACCAT<br>TCCTCCCAGTTTTCACCAAAGA
LDHA (lactate dehydrogenase A)	GCACCCAGTTTCCACCATGA<br>TTCAAACGGGCCTCTTCCTC
PDK1 (pyruvate dehydrogenase kinase, isozyme 1)	GAGAGCCACTATGGAACACCA<br>GGAGGTCTCAACACGAGGT
TFRC (transferrin transporter; CD71; TFR1)	ACCATTGTCATATACCCGGTTCA<br>GGCCTTTGTGTTATTGTCAGCAT
FTH1 (ferritin-H subunit)	CGAGGTGGCCGAATCTTCC<br>GTTTGTGCAGTTCCAGTAGTGA
FTL (ferritin-L subunit)	CAGCCTGGTCAATTTGTACCT<br>CCAGTTCGCGGAAGAAGTG
ACO1 (cytosolic aconitase = Iron Regulatory Protein 1)	GATATGGGCGCTTACCATTTTCG<br>GGCACACCCGTAAAGTCCTG
IREB2 (IRE-binding protein 2 = Iron Regulatory Protein 2)	CAATCCATCTGTCATGCTTGC<br>GGTAATACTCCACTTGAACTGAGG

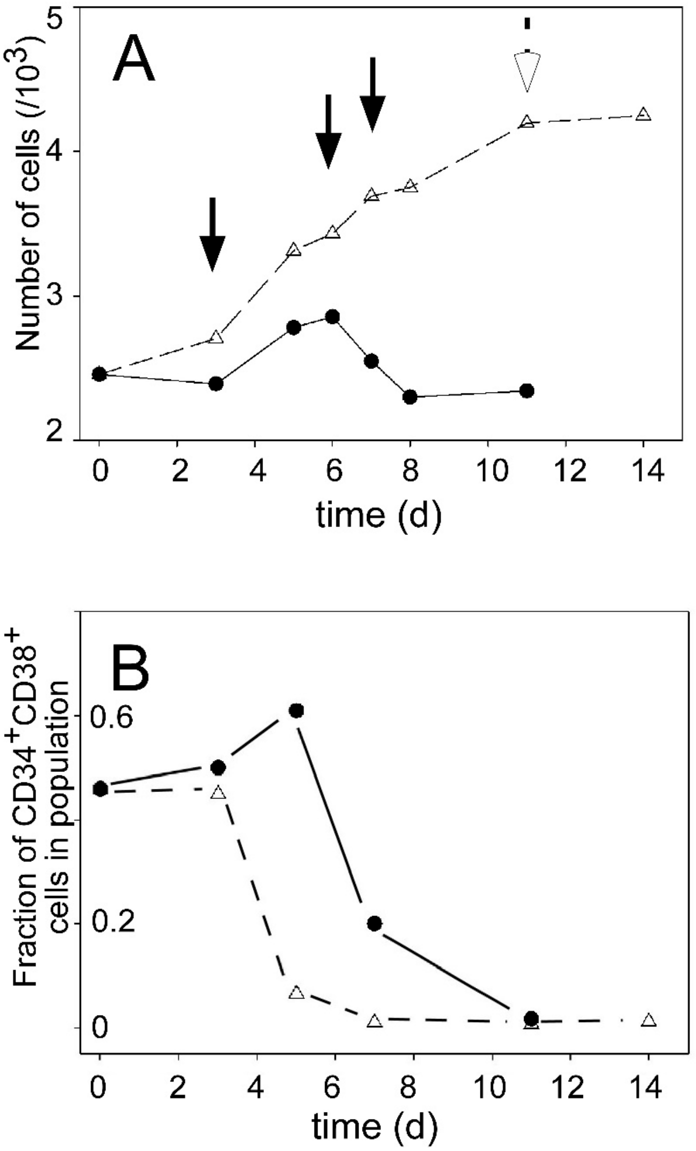

Figure S1. Evolution of cord blood CD34⁺ progenitors in synthetic media.

A. Growth of cord blood CD34⁺ cells in synthetic media. The number of cells was monitored as a function of time: these numbers were adjusted to the inoculum after considering withdrawals for analysis and media changes or additions (arrows). Cells were

incubated in the commercial medium (circles, solid line) or SMM-1 (empty triangles, dashed line) as detailed in Materials and Methods.

B. Fraction of the CD34$^+$CD38$^+$ population in the growth media in (A). The cells bearing these two markers were quantified by flow cytometry after labelling with CD34 Vioblue IgG2a and CD38 Phycoerythrin IgG2a. Their fractions are represented as circles for the commercial medium (solid line) or as empty triangles for SMM-1 (dashed line).

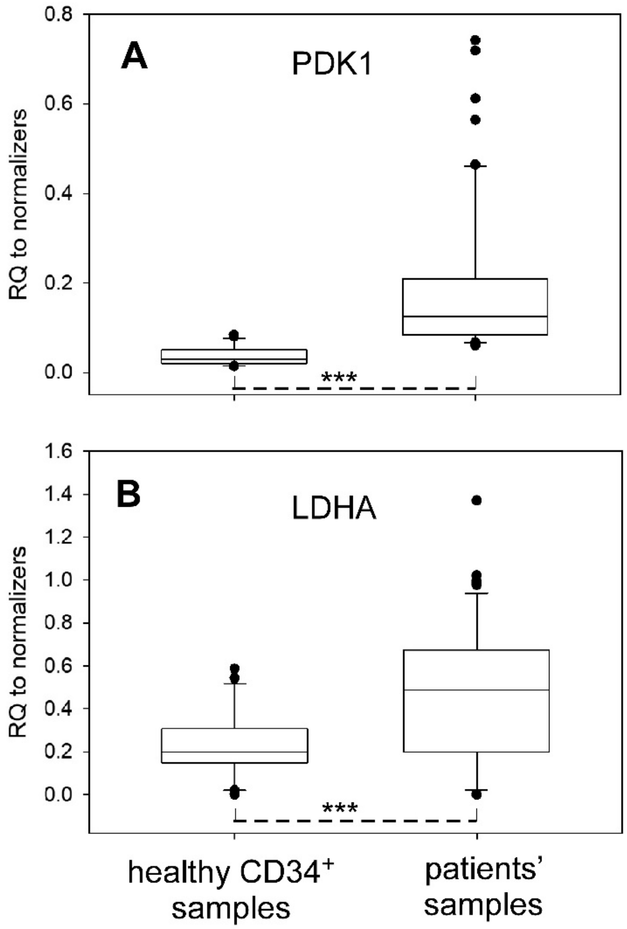

Figure S2. Expression of genes involved in energy metabolism. The abundance of the transcripts is relative to the 3 normalizers, RPLP0, HPRT, and ACTB. The boundaries of the boxes are the 25th and 75th percentiles with the internal lines marking the median values. The whiskers are the 90th and 10th percentiles. *** $P < 0.001$.

A. Amounts of PDK1 mRNA. The samples included 5 healthy donors, 1 providing cord blood and 4 bone marrow aspirates from adult donors, and 16 AML patients. The plot is the result of 23 and 52 measurements for healthy donors and patients, with means of 0.0365 (SD 0.0215) and 0.191 (SD 0.172), respectively. The *t*-test carried out with these data indicated significance for the difference between the two groups ($P < 0.001$).

B. Amounts of LDHA mRNA. The samples included 5 healthy donors, 2 providing cord blood and 3 bone marrow aspirates from adult donors, and 20 AML patients. The plot is the result of 24 and 58 measurements for healthy donors and patients, respectively. The *t*-test carried out with these data indicated significance for the difference between the two groups ($P < 0.001$).

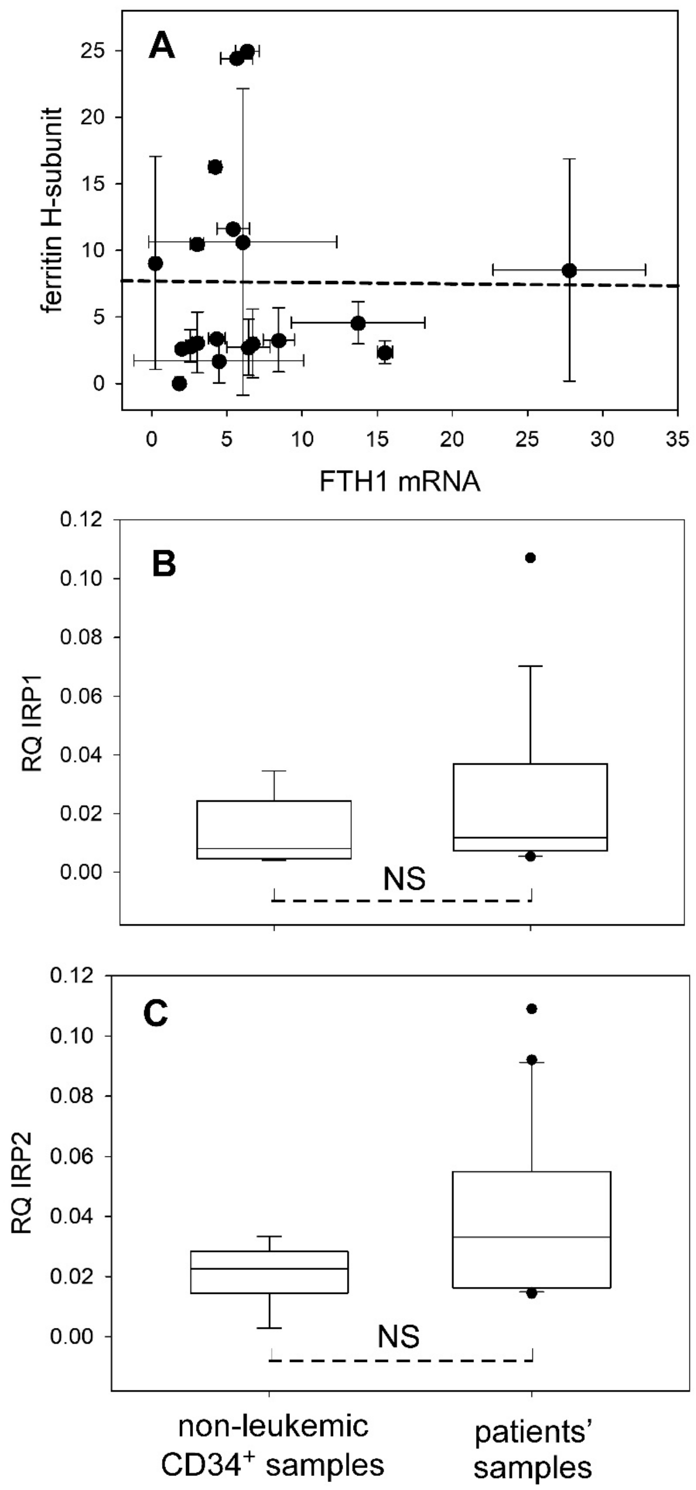

Figure S3. Transcription of genes of iron homeostasis in AML blasts.

A. Dependence of ferritin-H protein on the concentration of its mRNA. The values obtained by Western blots and RT-qPCR for each sample are plotted along the y- and x-axis, respectively.

B. Forest plot of IRP1 (ACO1) mRNA comparing non-leukemic $CD34^+$ progenitors and leukemic blasts. This plot is the result of 6 and 17 measurements for each group, respectively.

C. Forest plot of IRP2 (IREB2) mRNA comparing non-leukemic $CD34^+$ progenitors and leukemic blasts. This plot is the result 6 and 22 measurements for each group, respectively.

The boundaries of the boxes are the 25th and 75th percentiles with the internal lines marking the median values. The whiskers are the 90th and 10th percentiles. The abundance of the transcripts is relative to the 3 normalizers, RPLP0, HPRT, and ACTB. NS: not significant.

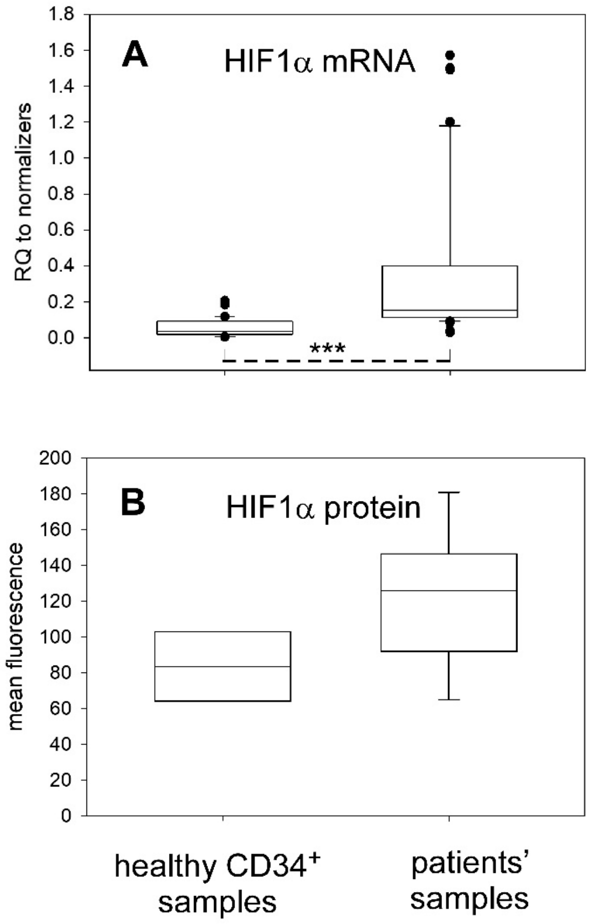

Figure S4. The HIF1α transcription factor in AML and healthy CD34^{+} cells**.**

A. The abundance of HIF1α mRNA is reported relative to the RPLP0, HPRT, and ACTB normalizers. The samples included 8 healthy donors (2 cord blood, 5 bone marrow aspirates from adult donors, and 1 from cytaphoresis rings) and 18 AML patients. The plot is the result of 33 and 51 measurements for healthy donors and patients, respectively.

B. The abundance of HIF1α protein was estimated by the fluorescence signal obtained after labeling and fluorescence activated cell separation. Nine AML patients' samples were combined in these experiments.

The boundaries of the boxes are the 25th and 75th percentiles with the internal lines marking the median values. The whiskers are the 90th and 10th percentiles. *** $P < 0.001$.
