# Supplementary figures and images for: Molecular Insight into Iron Homeostasis of Acute Myeloid Leukemia Blasts
Source: Int J Mol Sci. 2023 Sep 19;24(18):14307. doi: 10.3390/ijms241814307 (PMC10531764; doi:10.3390/ijms241814307)

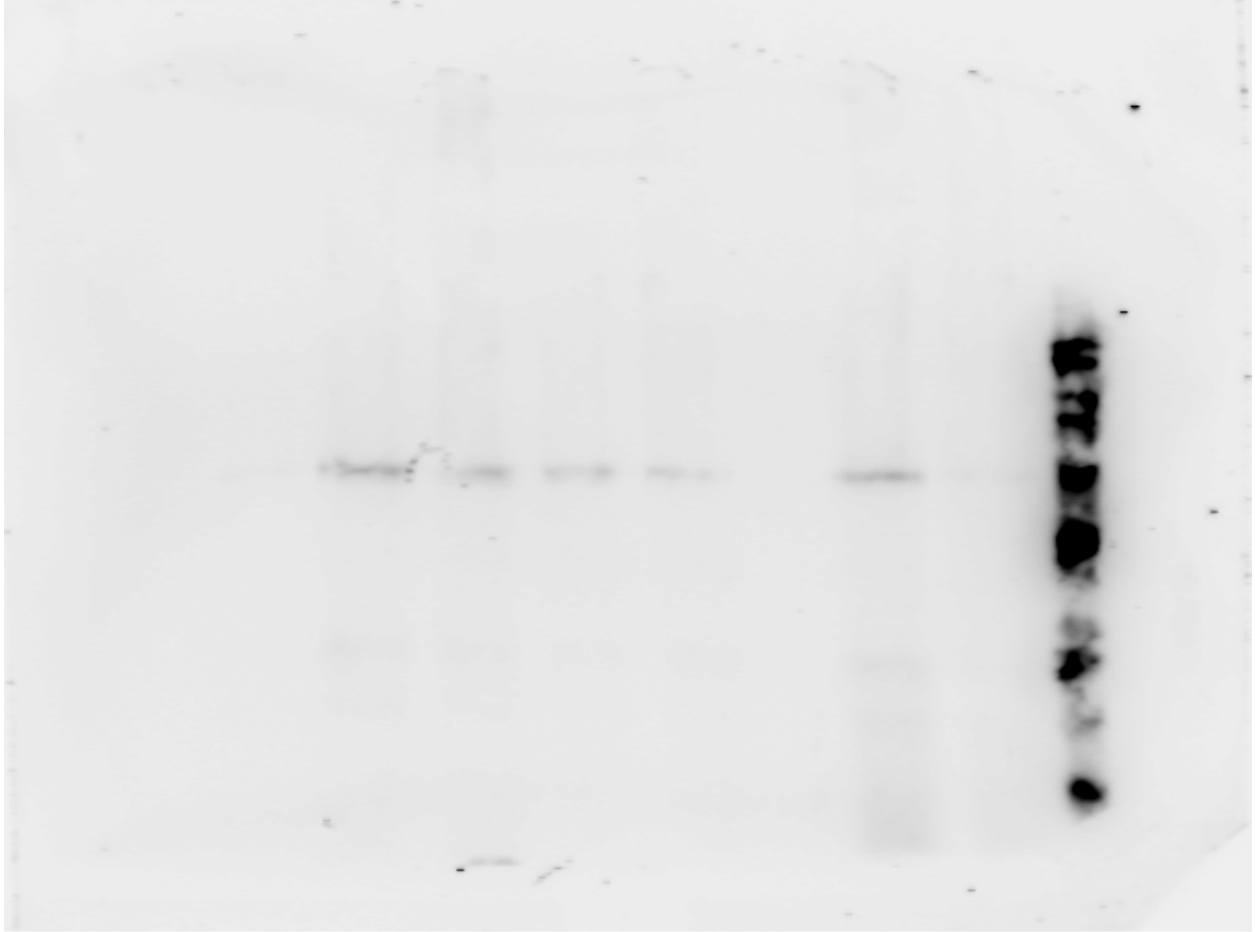

Western blot

Supplement: Supplementary file 1 [file ijms-24-14307-s001.zip › ijms-2594398-supplementary - wb.pdf]
